# Prenatal Exposure to Aluminum and Status of Selected Essential Trace Elements in Rural South African Women at Delivery

**DOI:** 10.3390/ijerph15071494

**Published:** 2018-07-15

**Authors:** Halina B. Röllin, Claudina Nogueira, Bukola Olutola, Kalavati Channa, Jon Ø. Odland

**Affiliations:** 1 School of Health Systems and Public Health, Faculty of Health Sciences, University of Pretoria, Private Bag X323, Pretoria 0001, South Africa; Claudina.Nogueira@up.ac.za (C.N.); bukola.olutola@gmail.com (B.O.); jon.oyvind.odland@uit.no (J.Ø.O.); 2 Environment and Health Research Unit, Medical Research Council, Johannesburg, 2193, South Africa; 3 Lancet Laboratories, Department of Analytical Chemistry, Johannesburg 2092, South Africa; kalavati.channa@lancet.co.za; 4 Department of Biomedical Technology, School of Health Sciences, University of Johannesburg, Johannesburg 2094, South Africa; 5 Institute of Community Medicine, University of Tromsø, Tromsø 9019, Norway

**Keywords:** aluminum, maternal serum and urine, essential trace elements, in utero exposure, birth outcomes

## Abstract

This study sought to evaluate the in utero exposure to aluminum and status of selected trace elements in South African women at delivery since aluminum is known to be toxic in all developmental stages even at low concentrations. Serum aluminum was negatively correlated with aluminum in urine, both uncorrected and corrected for creatinine, which suggests the retention of aluminum in body stores. Serum copper and zinc levels were found to be high in this study population. Serum copper levels were negatively correlated with aluminum in serum (β = −0.095; *p* = 0.05). There was a marginal negative correlation between aluminum levels in serum and manganese levels in whole blood (β = −0.087; *p* = 0.08). Copper levels in maternal serum were negatively correlated with birth weight and the length of neonates. There were a number of positive correlations between maternal characteristics and birth outcomes. Mothers who consumed root vegetables frequently appeared to be protected from aluminum retention and increased body burden since their serum aluminum levels were found to be significantly lower. The findings of the current study can be used as a baseline for further research on aluminum exposure and its associated interactions and outcomes in vulnerable populations.

## 1. Introduction

Aluminum (Al) is the third most abundant element in the Earth’s crust. Human exposure to Al from various sources is very common and increasing constantly [1]. Even though it has been estimated that, to meet the current global demand for Al, 11 kg of the metal must be produced yearly for every person on Earth, the use and efficiency of extraction by the Al industry cannot match that of the geochemical cycling of the metal since almost half of cast Al is destined to end up as waste [1]. Both newly extracted and waste Al has the potential to enter the biotic cycle. The consequences have already been manifested in the death of fish and trees in acidified surface waters and catchment areas, respectively, as well as limited plant growth on 30% of the Earth’s ice-free land [2,3].

Al is widely used in many industries such as engineering, food processing, in drinking water treatment as a flocculent, in pharmaceutical preparations, cosmetics and hygiene products, and in household implements such as Al cooking utensils. Inhalation, ingestion, and dermal absorption are the primary routes of exposure to Al in humans with ingestion being the most common. Individual exposure is also influenced by the geography, related anthropogenic activities, diets, use of Al-containing medications and dietary supplements, and use of Al saucepans and foils for cooking [4,5,6,7]. However, Al is still perceived to be a “safe” metal and there is no legislation limiting human exposure to Al. 

In humans, it has been shown that once the Al load exceeds the body’s excretory capacity, the excess is deposited in various tissues (bone, brain, liver, heart, spleen, and muscle), which has a negative impact on human health. Therefore, Al toxicity affects various systems and may manifest as encephalopathy, various bone disorders, proximal myopathy, increased risk of infection, increased left ventricular mass, decreased myocardial function, and microcytic anemia [8]. Al is known to be a neurotoxin and chronic exposure to even low levels of Al may lead to neurological disorders. As such, Al has been implicated in the etiology of Alzheimer’s disease and Parkinsonism-dementia even though the association between Al and the various types of dementia requires further investigation [9]. Adverse effects of non-occupational Al exposure in individuals with impaired renal function are well documented. These patients are typically exposed to Al through dialysate fluid or medicinal sources [10]. Therefore, Al plays a role in the etiology of many diseases with neurotoxicity being of particular concern. It is especially problematic in susceptible populations and in the developmental stages [11].

Although the knowledge base of Al toxicity has increased markedly in recent decades, very little is known regarding the reproductive toxicity of Al in humans [12]. To date, most studies have been performed in laboratory animal models. For example, in mice, it has been shown that intraperitoneal injection of Al sulphate at a dose of 200 mg/kg at 10 to 13 days of gestation not only lowered maternal weight but also produced behavioral and neurochemical alterations in newly born mice that persisted into adulthood [13]. This indicates that, at the perinatal stage, Al is highly neurotoxic and inhibits prenatal and postnatal brain development [13,14]. Other studies have shown that, although an excess intake of Al through the maternal diet during gestation and lactation did not produce maternal toxicity, it caused permanent neurobehavioral deficits in weaning mice and rats [15].

The research currently points to a possible Al role in human reproduction. High concentration of Al in human semen as well as the presence of Al in spermatozoa have been reported in patients with deteriorated semen quality, which suggests possible implications of Al in spermatogenesis and sperm count [16]. An analytical study on placenta tissues showed that Al was present in 95% of placenta body samples and 81% of placenta membrane samples, but only in 46% of umbilical cord samples, which indicates that the placenta acts as a partial barrier to Al exposure in utero [17]. The authors concluded that the developing fetus may still be vulnerable to cumulative Al exposure and that it is, therefore, important to establish reference ranges for Al levels in placental tissues [17]. Exposure to toxic metals including Al has been recently investigated as a potential cause of miscarriages [18]. This study identified increased levels of Al and other toxic metals in miscarried embryonic material and suggested some causative environmental and lifestyle factors. Prenatal exposure to Al during pregnancy has not been studied in any great depth in humans even though it is well established that the unborn infant is at an increased risk of Al toxicity in utero due to its immaturity related to anatomical and physiological factors [19]. In addition, Al overload has been demonstrated in neonates and pre-term infants requiring parenteral nutrition or intravenous fluid therapy [20,21,22]. Furthermore, neonatal Al exposure from parenteral nutrition in the high-risk pre-term infant may have adverse effects on bone health at a later stage as well as on short-term cognitive outcomes [20]. These studies all reinforce the well accepted vulnerability of the fetus, the neonate, and the developing infant to the toxicity of Al exposure. 

The extent of the toxic effects of Al can also depend on the status of essential elements such as copper (Cu), zinc (Zn), selenium (Se), manganese (Mn), and other elements since these trace metals are needed for enzymatic reactions, metabolic processes, and vital physiological and biochemical functions [23]. Therefore, their imbalance can be detrimental to health. For example, Cu is essential for hemoglobin synthesis, normal bone formation, and maintenance of myelin within the nervous system. Zn is required for protein and nucleic acid synthesis [24]. Increasingly, studies are demonstrating the importance of trace essential metals such as iron (Fe), Zn, and Cu in supporting a successful pregnancy [25]. Since essential elements are known to play significant roles in mediating the immune system and inflammatory responses, there may be an association between deficiencies of essential elements during pregnancy and the development of pregnancy complications mediated by oxidative stress and inflammation [26]. A recent study in a cohort of pregnant women in Australia found that a combined low Cu and Zn status was associated with a reduced risk of any pregnancy complications when compared with a high Cu and Zn status [26].

Since there is a scarcity of studies about the association between Al exposure in utero and the status of essential trace elements, the main aim of our study was to evaluate exposure levels to Al in the prenatal stage, which is measured in serum and urine samples from rural populations of South African women during delivery. The study also measured concentrations of selected essential elements (Cu, Zn, Se, and Mn) and their correlation with Al levels. Lastly, the possible associations between Al levels and certain factors (e.g., birth outcomes, socio-economic, housing, dietary, and lifestyle factors) were also investigated.

To the best of our knowledge, this is the first study that examines in utero exposure to Al and its effects on selected essential trace elements in a large cohort of South African delivering women. 

The current study is part of an international collaborative research being carried out under the umbrella of the Arctic Monitoring and Assessment Program (AMAP), which evaluates the outcomes of exposures to hazardous substances in vulnerable populations (such as pregnant women and their fetuses and infants). The AMAP research collaboration also compares the various study findings between the northern and southern hemispheres with the aim of informing policy and decision-making processes in the interests of environmental and public health.

## 2. Materials and Methods

### 2.1. Study Population

This cross-sectional study took place in four rural sites situated along the coastal regions of South Africa. The study participants were women admitted for delivery at the local maternity sections at local public hospitals. Women were informed about the study by admitting medical personnel on duty and given an information sheet about the study. Women who agreed to participate in the study signed an informed consent form and agreed to donate blood and urine samples before delivery. Participants agreed to answer a socio-demographic questionnaire by interview and consented to access and use of hospital birth outcome data (including maternal characteristics and neonate characteristics such as weight, length, and head circumference, gestational age, Apgar score), as well as birth complications, if any, for research purposes. The participation rate was high with 96% of approached women agreeing to take part in our study. In total, samples of blood and urine were collected from 450 women. Participation in the study was voluntary, confidentiality was assured, and participants were informed that they could withdraw from the study at any time.

### 2.2. Sample Collection

From each pregnant woman participating in the study, two samples of venous blood were collected using the Venoject sterile system and BD collection tubes. The process included one sample into (10 mL) EDTA-containing BD Vacutainer tube for whole blood analyses and one sample into a non-additive tube for serum analyses. The serum tubes were centrifuged and the serum was transferred to acid-washed polypropylene tubes using acid-washed plastic pipettes. For the collection of blood samples, non-powder gloves were used when handling and collecting samples. Midstream urine was collected into (30 mL) polypropylene acid-washed containers and, thereafter, decanted into polypropylene acid-washed tubes. The plastic containers had no metal caps or glued inserts and were not colored due to the metals found in dyes. All precautions to eliminate and prevent contamination at collection and sample preparation were applied throughout. Samples of collected whole blood, serum (post-centrifugation), and urine were stored at −20 °C and couriered in a frozen state to the National Institute for Occupational Health (NIOH) laboratory, Johannesburg, South Africa for analysis. The NIOH participates in a proficiency testing scheme for whole blood and urine.

### 2.3. Analytical Procedures for Al, Cu, Zn, Se in Serum and Mn in Whole Blood 

#### 2.3.1. Analysis of Al, Cu, Zn, Se in Serum

For the measurement of Al, Cu, and Zn in serum, 0.5 mL volumes of serum samples, internal standard solution containing ^45^Sc, ^72^Ge, (50 μL), 65% ultrapure nitric acid (50 μL) and ultra-pure water (4.4 mL) were pipetted into a polypropylene tube. The diluted serum samples were analyzed for element content using an Agilent 7500ce Inductively Coupled Plasma Mass Spectrometer (ICP-MS) with an Octopole Reaction System. The instrument was calibrated with calibration standards prepared using Seronorm^TM^ Trace Elements in serum (Sero Ltd., Billingstad, Norway) for matrix matching. For ^27^Al, ^45^Sc was used as the internal standard and analysis was performed in the no gas acquisition mode. ^63^Cu and ^66^Zn were measured in the helium gas mode with ^72^Ge used as the internal standard. Aliquots of each sample were analyzed in triplicate. The detection limits for Al, Cu, and Zn were 0.17 μg/L, 0.50 μg/L, and 0.40 μg/L, respectively. Seronorm^TM^ Trace Elements in serum (Sero Ltd., Billingstad, Norway) were analyzed with every analytical run in intervals of 10 samples for quality assurance of all element measurements.

For the Se assay, samples were diluted three-fold with equal amounts of a diluent solution (1.35% sodium chloride and 0.017% ammonium dihydrogen phosphate) and a palladium modifier solution (60% palladium 2000 mg/L in 0.5% Triton X-100). Se in serum measurements were carried out on a Thermo Scientific (Waltham, MA, USA) iCE3000 series spectrometer with graphite furnace and autosampler. A Se calibration curve was prepared by dilution of a 10 mg/L Se working stock solution so that the concentration ranged from 50 μg/L to 200 μg/L. ClinCheck serum control for trace elements level 1 and 2 were analyzed immediately after calibration and after every six samples for quality assurance of the Se determination. The detection limit for Se in serum was 6.5 μg/L.

#### 2.3.2. Analysis of Mn in Whole Blood

For the measurement of Mn in whole blood, 0.5 mL of the blood sample was pipetted into a polypropylene digestion tube, which is followed by the addition of 65% ultrapure nitric acid (1 mL). The mixture was digested at 90 °C for 2 h. Once cooled, 70 µL internal standard solution containing ^45^Sc was added and further diluted with ultrapure water to a final volume of 7 mL. The digested blood samples were analyzed using an Agilent 7500ce (ICP-MS) with an Octopole Reaction System. The instrument was calibrated with calibration standards prepared using Seronorm^TM^ Trace Elements in whole blood level 1 for matrix matching. The analysis was performed in a helium acquisition mode. Aliquots of each sample were analyzed in triplicate. The detection limit for Mn was 0.07 μg/L. Two certified reference controls known as Seronorm^TM^ Trace Elements in whole blood levels 1 and 2 (Sero Ltd., Billingstad, Norway) were analyzed with every analytical run in intervals of 10 samples for quality assurance of all element measurements.

#### 2.3.3. Analysis of Al in Urine

Urine samples (1 mL volumes) were acidified with 0.1 mL of 65% ultrapure nitric acid (Fluka, Munich, Germany). An internal standard solution containing ^72^Ge was added (50 μL) to all samples, reagent blanks, reference controls, blank urine collection tubes, and calibration standards. The measured solution held 5 mL (5 times sample dilution) with deionized water. Urinary Al levels were measured in the no gas acquisition mode. The percentage recovery, when using certified controls (Lyphochek level 1 and 2), was 95.1% and 97.6% for level 1 and 2, respectively. The detection limits for urinary Al was 1.71 (SD 0.41) μg/L.

#### 2.3.4. Analyses of Creatinine in Urine

The creatinine concentration in urine samples was determined by using the Jaffé rate method and an automated Roche Cobas 111 analyzer. Urine samples were dispensed into the Cobas cups, which were automatically injected in a reaction cell containing an alkaline picrate solution (Cobas 111 creatinine Jaffé CREJ2 reagent 1 and 2). The sample combined with the reagent to produce a yellow-orange colored complex (alkaline-picrate creatinine complex), which is directly proportional to the creatinine concentration in the sample. The Cobas Calibrator for automated systems, Ref 10759300, was used for the assay. Certified controls, ‘Liquichek Urine Chemistry Control’, level 1 and 2 (Bio-Rad, Hercules, California, USA) were run before and after every 20 samples.

### 2.4. Covariates

Covariate information was obtained during the questionnaire-based interview and from medical records. Maternal weight and height were recorded at the hospital on admission. From the medical records, the neonate characteristics retrieved include birth weight (g), birth length (cm), head circumference (cm), and gestational age (weeks). Pre-term labor was defined as mothers giving birth at less than 37 weeks of gestational age. Education was categorized as no education to completed primary school, completed secondary school, and any level of tertiary education attained. Maternal tobacco smoking during pregnancy was defined as yes or no. Exposure to environmental tobacco smoke (ETS) was defined as exposure to tobacco smoke from smoking by others in the household. A binary classification was used for exposure to indoor smoke from the burning of fossil fuel (wood and coal) for the purpose of heating or cooking as well as separating study participants into those exposed to fossil fuel and those not exposed (for example, those using electricity). Dietary questions relating to the intake of proteins, carbohydrates, dairy products, tea, coffee, bottled water, fruits, and vine, root and leafy vegetables were assessed and classified as daily, at least once a week, and seldom (both for pre-pregnancy and during pregnancy).

### 2.5. Statistical Analyses

The statistical analyses were performed using STATA (StataCorp, 2013. Stata Statistical Software: Release 13. College Station, TX, USA: StataCorp LP). Al was detected in all serum and urine samples. Bivariate analyses between maternal serum Al exposure and covariates were evaluated by using the Spearman’s correlation coefficient. The distribution of maternal serum Al was skewed and was square-root-transformed after exploring the best fit for transformation. Multivariate linear regression was carried out using a backward deletion approach by starting with a full model of factors significantly associated with natural square-root-transformed maternal serum Al levels in the univariate analysis. All statistical tests were two-tailed and statistical significance was set at *p* < 0.05.

### 2.6. Ethical Considerations

The work was carried out in accordance with The Code of Ethics of the World Medical Association (Declaration of Helsinki). Ethics approval for the study was obtained from the Human Research Ethics Committee of the University of Witwatersrand in Johannesburg (Protocol no. M10742), and from the relevant provincial Departments of Health. In addition, CEOs of the respective hospitals had to confirm that he/she allowed the research work to proceed. Identical procedures were followed in terms of obtaining consent from participants. Confidentiality was maintained by assigning identification numbers to all study participants. During the informed consent process, it was emphasized that participation was voluntary and could be withdrawn at any time.

## 3. Results

### 3.1. Participant Characteristics

The background characteristics of study participants are presented in Table 1. Most of the mothers were single and of African Black ethnicity. They were well educated with one-third obtaining tertiary level education. Many resided in formal housing with most of the households having access to potable municipal tap water but 15.3% of participants had to rely on rivers and borehole sources for drinking water. Self-reported smoking prevalence and alcohol consumption was very low.

### 3.2. Obstetric and Birth Outcomes

Table 2 shows the descriptive data for obstetrics and birth outcomes. The average weight [mean, (SD)] of the mothers before delivery was 72.1 (13.1) kg and the average birth weight of their infants was 3055 (484.8) g, which ranged from 1300 g to 5150 g. The average birth length was 49.3 (3.5) cm and head circumference was 34.8 (1.8) cm, which ranged from 25 cm to 47 cm. The average gestational age was 38.1 weeks, which ranged from 29 to 47 weeks. A total of 50.5% of neonates were male. Most of the infants (76.27%, *n* = 315) had an Apgar score of 9 at 1 min while 93.95% (*n* = 388) scored 10 as an Apgar score at 5 min. Almost 45% of women were primiparous and more than 55% had one or more children.

### 3.3. Concentration of Serum Al, Urinary Al, and Selected Essential Elements

Concentrations of Al, Cu, Zn, and Se in serum, Mn in whole blood, and Al in urine (uncorrected and creatinine corrected) within the study cohort at delivery are shown in Table 3. The average maternal serum concentration of Al was 10.1 (7.95) µg/L with geometric mean (GM) of 6.79 µg/L (95% CI: 6.12–7.53). The mean concentration of Al in urine was 18.1 (14.9) µg/L with GM of 13.10 µg/L (95% CI: 11.97–14.35). After correction for creatinine, the mean Al levels in urine were 21.4 (20.03) µg/g and creatinine with GM of 15.09 µg/g creatinine (95% CI: 13.75–16.55).

GM for Cu levels in serum was 2433 µg/L (95% CI: 2379–2488,) and for Zn serum 497.8 µg/L (95% CI: 484.5–511.4). Se concentration in serum (GM) was 62.82 µg/L (95% CI: 61.08–64.61) and GM of Mn in whole blood was 15.85 µg/L (95% CI: 15.28–16.45).

### 3.4. Association between Serum Al and Selected Essential Elements

A bivariate analysis was performed to determine the association between serum Al levels and selected essential trace elements (Table 4). Negative correlations were shown between maternal serum Al and serum Se as well as between maternal serum Al and Mn in whole blood. The study found a positive correlation between maternal serum Al and maternal serum Zn. However, the correlation was not statistically significant. The only significant correlation was between the maternal serum Al and the maternal serum Cu, which was found to be negative (β = −0.095, *p* = 0.05).

### 3.5. Association between Serum Al, Selected Essential Elements, Maternal Covariates, and Infant Anthropometry

A negative correlation was found between maternal serum Al and maternal urine Al (rho = −0.222, *p* = 0.001) as well as for creatinine corrected urine Al (rho = −0.266, *p* < 0.001). A significant negative correlation was found between maternal serum Al and serum Cu (rho = −0.187, *p* = 0.007). Similarly, a negative correlation was evident between the maternal serum Al and the maternal blood Mn (rho = −0.168, *p* = 0.015) (Table 5). Levels of Al, Zn, and Se in maternal serum and Mn in whole blood did not seem to affect anthropometry of infants in our study cohort. However, maternal serum Cu was negatively correlated with infant birth weight (rho = −0.144, *p* = 0.037) and birth length (rho = −0.152, *p* = 0.028). Parity was positively correlated with birth weight (rho = 0.171, *p* = 0.013). 

Furthermore, a positive correlation was observed between maternal age and birth weight (rho = 0.241, *p* < 0.001), maternal weight and birth weight (rho = 0.175, *p* = 0.011), and maternal weight and birth length of infants (rho = 0.150, *p* = 0.030). There was also a positive correlation between the maternal height and birth weight (rho = 0.237, *p* < 0.001) (Table 5).

### 3.6. Univariate and Multivariate Analysis of Association

#### 3.6.1. Univariate Analysis

In the univariate analysis (Table 6), no association was found between serum maternal Al and essential elements. Maternal serum Al levels were not associated with the neonate gender. Mothers who did not smoke during pregnancy were less likely to have elevated maternal serum Al levels when compared to mothers who smoked during pregnancy (β = −0.542, 95% CI: −0.808 to −0.277, *p* ≤ 0.001). Mothers who ate tinned meat almost every day before pregnancy were more likely to have elevated serum Al levels than mothers who seldom ate tinned meat before pregnancy (β = 0.531, 95% CI: 0.180 to 0.882, *p* = 0.003). Similarly, mothers who ate tinned meat almost every day during pregnancy were more likely to have higher levels of serum Al than mothers who seldom ate this type of meat during pregnancy (β = 0.373, 95% CI: 0.011 to 0.735, *p* = 0.03). 

#### 3.6.2. Multivariate Analysis

In the final multivariate analysis (shown in Table 6), the factors associated with maternal serum Al were identified. Mothers of other races were most likely to have lower serum Al levels compared to African Black mothers (β = −0.662, 95% CI: −1.06 to −0.263, *p* = 0.001). Mothers using paraffin as a source of heating in the home were less likely to have high maternal serum Al levels when compared to mothers using electricity (β = −0.539, 95% CI: −1.044 to −0.035; *p* = 0.036). However, there was no significant difference in the levels of maternal serum Al in mothers using gas/wood/coal and electricity for heating. Mothers who ate root vegetables during pregnancy almost every day were less likely to have high maternal serum Al levels compared to those who ate these vegetables rarely or at least once per week. The study did not find any significant correlations between serum Al concentrations and other dietary intakes including beverages.

Those who reported that their houses were not regularly sprayed with insecticides as part of a malaria control program were less likely to have higher serum Al levels when compared to those who had their houses sprayed regularly.

## 4. Discussion

The current study has assessed exposure to Al in utero at delivery in a rural population of pregnant women in South Africa. The study also measured selected essential trace elements (Cu, Zn, Se, and Mn) and identified the potential effects of contributing socio-economic and lifestyle factors on Al levels. 

Al was detected in all serum and urine samples. The study has found statistically significant negative correlations between Al levels in maternal serum and Al levels in maternal urine (both uncorrected and corrected for creatinine). This finding may indicate limited Al excretion in the study population and subsequent Al deposition in tissues. It is very difficult to postulate if Al levels in serum in this study population are high enough to result in toxicity since, at present, there are no referenced serum Al levels for pregnant women [27].

In terms of reference values, early reports published from 1980 to 1985 showed mean serum Al values in healthy subjects ranging from 1.9 μg/L to 10.3 μg/L and an overall median of 6.2 μg/L [28]. A review of further reports published from 1986 to 1992 showed a range of 0 μg/L to 5.97 μg/L for Al in serum with an overall median of 3.25 μg/L [29].

Presently, according to the Agency for Toxic Substances and Disease Registry (ATSDR) of the US Department of Health and Human Services, Public Health Services, Atlanta, GA, USA, Al serum levels in healthy individuals who are not pregnant range from 1 μg/L to 3 μg/L. Serum Al levels above 50 μg/L are considered toxic [30].

The mean Al serum level in pregnant women at delivery in our study was 10.1 μg/L with an overall median of 6.79 μg/L, which is above the ATSDR guidance. These results are comparable to the 13.92 ± 14.09 μg/L concentrations of Al in plasma, which were measured in a cohort of pregnant women practicing the ancestral cultural custom of ingestion of earth (geophagy) in French Guiana [31]. A comparable study measured Al in maternal blood in Japanese women and the Al levels reported were 7.83 μg/L [32]. In most of the reported studies, a direct comparison with our results and the measured umbilical serum Al in neonates is not possible. For example, Sedman et al., in 1985, reported serum Al levels of mean 5.17 μg/L in neonates and a similar study in 1989 reported serum Al levels of 8 μg/L to 12 μg/L [33,34]. The study by Rahbar et al., in 2015, measured Al in the umbilical cord blood as well as Mn levels in Jamaican newborns. The findings indicated mean concentrations of 10.9 (9.2) μg/L and 43.7 (17.7) μg/L for Al and Mn, respectively [35].

Furthermore, significant differences in Al levels were reported at different gestational stages. Bougle et al., in 1992, investigated plasma Al concentrations in infants born at different gestational ages. The mean plasma Al level in pre-term infants born at gestational ages between 28 to 32 weeks, pre-term infants born at gestational ages between 33 to 36 weeks, and full-term (mean 39 weeks) infants were 13.2 μg/L, 10.5 μg/L, and 7.8 μg/L, respectively [36]. Urinary Al concentrations in the present study are negatively correlated with Al in serum. This may be an indication of retention of Al in storage organs.

Recently, awareness has increased with regard to the consequences of overexposure to biologically reactive Al at both pre-natal and post-natal stages. This overexposure may impact the health of the developing infant and increase infant susceptibility to a range of diseases [15]. Additionally, since 2004, the Food and Drug Administration (FDA) has recommended restricting daily Al administration to 5 μg/kg of body weight for parenteral nutrition and intravenous fluid therapy products in neonates and pre-term infants precisely because of the well-established adverse health effects of Al [37]. 

During pregnancy, fetal exposure to Al is influenced not only by the maternal environment and diet but also by maternal use of Al-containing antacids and other Al-containing medications. In addition, it has been shown that concomitant consumption of citrate-containing beverages by pregnant women significantly increases absorption of Al in the gut [38,39]. The same Al absorption effect has been shown in hemodialysis patients [40]. After birth, infants continue to ingest Al from human breast milk or infant formulas. Therefore, Al content in human breast milk ranges from 15 μg/L to 30 μg/L. The content of Al in infant formulas especially in soy-based preparations has been found to be many times higher. It was found to range from 100 μg/L to 900 μg/L [37]. In short, exposure to Al during the developmental stages produces toxicity that may impair fetal growth or development by interfering with the GTPase cycle, the free radical-mediated cytotoxicity, lipid peroxidation, and changes in serum essential elements [41,42]. Therefore, the vulnerability of infants to early exposure of Al points to an urgent need to reduce the Al content of infant formulas to as low a level as possible [43].

The current study also measured the levels of essential trace elements in delivering women. The concentrations of Cu and Zn in serum exceeded reference values for the general population [27]. In contrast, levels of Se in serum in our study population were similar to those reported for the general population while the concentrations of Mn in whole blood were higher [44]. The recent Jamaican study already mentioned measured Al and Mn concurrently in the umbilical cord blood and reported higher levels of Mn (43.7 μg/L) [32].

Most of the studies that investigated concentrations of essential trace elements in populations of pregnant women elsewhere in the world did not measure associated Al levels. Therefore, direct comparisons cannot be made with the results of our study, but the levels of the trace elements can be used as indicators. 

A Turkish study found a significant low level of serum Se and Zn and a high level of serum Cu in pregnant women when compared to reference populations of non-pregnant women and men [45]. In contrast, our study found high serum levels for both Cu and Zn, and the level of serum Se was comparable to reference populations. The ranges identified in our current study are reasonably aligned with the values obtained for Se, Zn, and Cu in a study carried out in Arctic Canada while, in the latter study, the biological samples were plasma and not serum [46]. A cohort of pregnant women in Pakistan found Zn levels to be higher in the pregnant population when compared to non-pregnant controls [47]. A similar study, comparing populations of pregnant women in Malawi and the Philippines [48], found decreased levels of Se in plasma samples from the Malawi study population when compared to a similar population from the Philippines, which is mainly attributed to reduced dietary intake and differing soil compositions. However, another study, in a USA cohort near a mining impacted site, investigated Mn levels in paired maternal and cord blood samples at delivery. This study found that the median blood Mn levels were lower than a comparable cohort in China, but higher than similar study cohorts reported elsewhere including Germany, France, Canada, and South Africa [49]. It has been postulated that competition between Al and essential trace elements during pregnancy may be one of the possible mechanisms for explaining adverse reproductive outcomes related to Al toxicity since it is well recognized that oral Al exposure during pregnancy can produce significant changes in the tissue distribution of a number of essential elements [50]. This concept has been investigated mainly in patients undergoing hemodialysis [Navarro et al., 1989 [51]] and occupationally exposed workers [52]. Our previous study on foundry workers found an increase in serum Al levels at low exposures to Al dust (1 mg Al/m^3)^, an incomplete excretion of Al in the urine but a significant decrease in Cu and increase in Zn serum levels [53].

However, very few studies to date have investigated the impact of Al exposure on the status of essential trace elements in vulnerable populations such as pregnant women and their developing fetuses. This is particularly the case in the southern hemisphere and in developing countries where almost no information is available. The demand for energy and nutrients is increased during pregnancy especially for micronutrients such as Cu, Zn and Se, which are involved in numerous biological processes for the maintenance of life [54]. Since most toxic and essential metals share chemical properties, it has been suggested that a number of metabolic interactions takes place between essential and toxic metals (like Al), which could reduce the levels of essential metals or increase the health risks associated with toxic metals [23] through the subsequent impact on cellular enzymatic and metabolic processes [52]. Similarly, it is well recognized that high maternal intake of Al can result in the altered metabolism of trace elements in the offspring [50]. By altering the levels of essential trace elements, Al may introduce an added dimension to its well established toxicity.

Our current study has found statistically significant negative correlations between Al and Cu levels in maternal serum as well as between Al levels in maternal serum and Mn levels in maternal whole blood. The latter finding warrants further investigation. For example, it is widely accepted that Mn supplements should not be taken together with Al-containing medications such as antacids because of the known interaction between the two metals. As an essential metal, Mn is involved in the formation of bone and in the metabolism of amino acids, cholesterol, and carbohydrates. It is an enzyme activator and a component of metallo-enzymes. It is also involved in the maintenance of healthy reproductive, nervous, and immune systems [55]. An inverted U-shape relationship between maternal Mn levels and infant birth weight has been shown by Zota el al. [56]. No correlation was found between Al and Zn or between Al and Se in maternal serum. Elevated Cu serum levels are known to cause Zn levels to deplete even though this is not the case with the population of this study, which also shows high concentrations of serum Zn and a positive correlation between Al and Zn despite not being statistically significant. Cu deficiency is known to provoke iron deficiency and microcytic anemia [52]. In addition, maintaining a healthy Cu to Zn ratio is extremely important for healthy brain function since elevated Cu levels can alter the activity of dopamine and nor-epinephrine [24]. In the current study, the concentrations of Al in maternal serum and urine did not affect infant anthropometry outcomes. However, this study found that maternal serum Cu levels were negatively correlated and statistically significant in terms of two of the infant anthropometry measures, which are infant birth weight and infant birth length. Positive and statistically significant correlations were also found between the age and weight of the mother and the birth weight and length of the infant. A positive and statistically significant correlation was also found between the infant birth weight and both parity and maternal height.

Both in univariate and multivariate analysis, no associations were found between maternal Al levels and essential elements measured. Maternal serum Al levels were not associated with the neonate gender. Mothers who consumed root vegetables frequently before and during their pregnancies appear to be protected from Al retention and increased body burden since their serum Al levels were found to be lower (statistically significant). Al levels in plant foods reflect the Al content of the soil and water from where they were grown. There may be considerable variation in the Al concentrations of fresh foods depending on environmental factors, soil contamination, or failure of adequate washing of the food products. In the absence of contamination, the Al concentrations in fresh vegetables range from around 0.5 to 3.0 mg per kg of weight in a number of countries despite the fact that some herbs, teas, and spices are known to absorb and retain much higher concentrations of Al [57]. In the main, unprocessed foods like fresh fruits, vegetables, and meat contain very little Al and exposure through ingestion is more likely to be brought about by external contamination such as the use of Al pots for cooking. The findings observed in our study may indicate that there is very little Al in the environment where the vegetables were grown and that the study group may not have made use of Al cookware on a daily basis.

Mothers who lived in houses that were not regularly sprayed with malaria control chemical agents were found to have lower serum Al concentrations (statistically significant) than those whose homes were regularly sprayed. A number of pesticide preparations are known to contain toxic metals, which, in turn, can be absorbed into the environment where they persist and accumulate in the soil and water, which ultimately impacts human and environmental health.

## 5. Conclusions

The present study has evaluated the in utero exposure to Al in a large cohort of rural South African women and can be used as a baseline for further investigations. Scientific consensus indicates that Al is toxic in all developmental stages and may produce irreversible health effects even at low levels. Since Al is known to cross the placental barrier, pregnant women should be advised against consumption of Al-containing antacids and the use of Al utensils for cooking. They should also be informed about avoiding post-natal exposure of their children to Al. 

The current study has also addressed potential metabolic interactions between Al and essential trace elements during pregnancy and our findings indicate a significant correlation between low Al levels and some of the selected essential elements, which indicates competition between Al and essential elements for common binding sites. It is widely accepted that essential trace elements are critical for fetal development even though their associated mechanisms in pregnancy are not fully understood. 

From a public health perspective, further investigations are required for preventing the detrimental health effects of Al exposure and its associated interactions and outcomes in vulnerable populations such as pregnant women and their developing fetuses. Limitations identified for this study include the cross-sectional design and a failure to interrogate the intake of antacids during pregnancy as part of the questionnaire administered to the study participants. Although our study cohort was large, we collected samples of blood and urine only at the delivery stage. Collections during the first and second trimesters were not possible due to financial constraints and because large distances had to be traversed to reach the study participants at various rural sites across South Africa. 

## Figures and Tables

**Table 1:** Socio-economic characteristics of study population.

Characteristic	*N* = 450
Mothers’ characteristics	
Age (years) [mean, (SD)]	24.8 (6.2)
Marital status (*n*, %)	
Married	73 (16.5)
Single	294 (66.5)
Co-habiting	71 (16.1)
Education (*n*, %)	
None/Primary	51(12.2)
Secondary	223 (52.1)
Tertiary	153 (35.8)
Race/ethnicity (*n*, %)	
African Black	383 (86.7)
Others	51 (11.5)
Percentage unemployed (*n*, %)	361 (81)
Ownership of home (*n*, %)	
Owned	396 (89.2)
Rented	48 (10.8)
Housing type (*n*, %)	
Formal housing	361 (81.7)
Flat	13 (2.9)
Backyard dwelling	9 (2.0)
Informal house (shack)	43 (9.7)
Other	16 (3.6)
Fuel used for cooking (*n*, %)	
Electricity	287 (64.8)
Paraffin	31 (7.0)
Gas/Wood	125 (28.2)
Fuel used for heating (*n*, %)	
Electricity	220 (50.1)
Paraffin	27 (6.2)
Gas/wood/coal	80 (18.2)
None	112 (25.5)
Source of drinking water (*n*, %)	
Indoor tap	111 (25)
Outdoor tap	265 (59.7)
Other (borehole and river)	61 (15.3)
Perception that air quality is good in the neighborhood (*n*, %)	358 (80.8)

**Table 2:** Obstetric and birth outcomes.

Characteristic/Parameter	Total (*N* = 450)	Range
Maternal age (y)	24.8 (6.2)	14–49
Maternal weight (kg)	72.1 (13.1)	41–128
Maternal height (cm)	158.1 (9.6)	123.4–176
Gestational age (weeks) [mean, (SD)]	38.1 (1.9)	29–47
Birth weight (g) [mean, (SD)]	3055 (484.8)	1300–5150
Birth length (cm) [mean, (SD)]	49.3 (3.5)	34–57
Head circumference (cm) [mean, (SD)]	34.8 (1.8)	25–47
Apgar score 1 min [mean, (SD)]	9.1 (8.9)	2–10
Apgar score 5 min [mean, (SD)]	9.9 (0.7)	3–10
Sex (% male)	50.5	
Parity (%)		
0	44.9	
1+	55.1	

**Table 3:** Concentration of Al in serum and urine, Cu, Zn, and Se in serum and Mn in whole blood in South African delivering women.

Element	*N* *	Mean (SD)	Range	GM	95% Confidence Interval
Al serum (ug/L)	425	10.1 (7.95)	0.25–59.42	6.79	6.12; 7.53
Al urine (ug/L)	318	18.1 (14.92)	2.21–106.3	13.10	11.97; 14.35
Al urine (ug/g creatinine)	318	21.4 (20.03)	1.45–28.1	15.09	13.75; 16.55
Cu serum (ug/L)	447	2496 (539)	204.8–458	2433	2379; 2488
Zn serum (ug/L)	446	518.8 (156.7)	127.7–1810	497.8	484.5; 511.4
Se serum (ug/L)	444	65.69 (20.4)	10–182.5	62.82	61.08; 64.61
Mn blood (ug/L)	441	17.13 (7.03)	4.49–3.88	15.85	15.28; 16.45

*N* *—total number of samples analyzed may differ.

**Table 4:** Spearman’s rank correlation coefficient (*p*-value) of association between maternal serum Al and essential elements (Cu, Zn, and Se in serum and Mn in blood) at delivery (*n* = 416).

Element	β	*p*-Value
Copper (serum)	−0.095	0.05 *
Zinc (serum)	0.035	0.48
Selenium (serum)	−0.059	0.23
Manganese (whole blood)	−0.087	0.08

* Statistically significant.

**Table 5:** Spearman’s rank correlation coefficient (*p*-value) of association between maternal serum Al, the status of selected essential elements, maternal covariates, and infant anthropometry.

Covariates	Birth Weight (g)	Birth Length (cm)	Birth Head Circumference (cm)	Maternal Serum Al (ug/L)
Maternal serum Al	0.087 (0.207)	−0.057 (0.409)	0.128 (0.064)	-
Maternal urine Al	0.037 (0.593)	0.071 (0.305)	0.052 (0.455)	−0.222 (0.001) *
Maternal urine Al/Creatinine corrected	0.020 (0.775)	0.024 (0.731)	0.055 (0.426)	−0.266 (<0.001) *
Maternal serum Cu	−0.144 (0.037) *	−0.152 (0.028) *	−0.067 (0.335)	−0.187 (0.007) *
Maternal serum Zn	−0.047 (0.499)	−0.013 (0.850)	0.051 (0.464)	0.007 (0.917)
Maternal serum Se	0.114 (0.099)	0.094 (0.175)	0.075 (0.279)	−0.022 (0.750)
Maternal blood Mn	−0.123 (0.076)	−0.024 (0.730)	−0.095 (0.172)	−0.168 (0.015) *
Age	0.241 (<0.001) *	0.207 (0.003) *	0.053 (0.448)	−0.076 (0.270)
Parity	0.171 (0.013) *	0.124 (0.072)	0.053 (0.444)	0.006 (0.930)
Maternal weight	0.175 (0.011) *	0.150 (0.030) *	0.043 (0.537)	−0.010 (0.882)
Maternal height	0.237 (<0.001) *	0.069 (0.320)	0.052 (0.452)	0.001 (0.988)

* Statistically significant.

**Table 6:** Factors predicting Al levels in maternal serum: univariate and multivariate analyses.

Characteristics	Univariate Analysis			Multivariate Analysis		
	β (Unadjusted)	*p*-Value	95% Conf. Interval	β (Adjusted)	*p*-Value	95% Conf. Interval
Maternal urine Al	−0.133	0.062	−0.273 to 0.007	-	-	-
Maternal urine (Al/creatinine corrected)	−0.152	0.030	−0.288 to −0.015	-	-	-
Maternal serum Se	−0.144	0.478	−0.542 to 0.254	-	-	-
Maternal serum Zn	0.163	0.424	−0.238 to 0.564			
Maternal serum Cu	−0.427	0.084	−0.911 to 0.058	-	-	-
Maternal blood Mn	−0.250	0.081	−0.530 to 0.031	-	-	-
Gender of baby						
Male	Reference					
Female	−0.034	0.780	−0.272 to 0.205	-	-	-
Smoked during pregnancy						
Yes	Reference					
No	−0.542	<0.001	−0.808 to −0.277	-	-	-
Ate tinned meat before pregnancy						
Seldom	Reference					
At least once/week	0.136	0.414	−0.191 to 0.464	-	-	-
Almost every day	0.531	0.003	0.180 to 0.882	-	-	-
Ate tinned meat during pregnancy						
Seldom	Reference					
At least once/week	−0.017	0.921	−0.352 to 0.318	-	-	-
Almost every day	0.373	0.043	0.011 to 0.735	-	-	-
Race/Ethnicity						
African Black	Reference					
Other	−0.760	<0.001	−1.41 to −0.379	−0.662	0.001	−1.060 to −0.263
Fuel used for heating						
Electricity	Reference			Reference		
Paraffin	−0.429	0.106	−0.949 to 0.092	−0.539	0.036	−1.044 to −0.035
Gas/Wood/Coal	0.155	0.339	−0.164 to 0.473	−0.199	0.236	−0.530 to 0.131
None	0.343	0.019	0.057 to 0.629	−0.260	0.130	−0.597 to 0.077
Ate root vegetables during pregnancy						
Seldom/At least once/week	Reference			Reference		
Almost every day	−0.481	0.001	−0.754 to −0.207	−0.383	0.009	−0.668 to −0.098
House regularly sprayed by malaria control program						
Yes	Reference			Reference		
No	−0.727	<0.001	−0.992 to −0.463	−0.664	<0.001	−0.990 to −0.339
